# Kir6.1 improves cardiac dysfunction in diabetic cardiomyopathy via the AKT‐FoxO1 signalling pathway

**DOI:** 10.1111/jcmm.16346

**Published:** 2021-02-06

**Authors:** Jinxin Wang, Jing Bai, Peng Duan, Hao Wang, Yang Li, Qinglei Zhu

**Affiliations:** ^1^ Department of Cardiology Chinese PLA General Hospital Beijing China; ^2^ Department of Geriatric Cardiology Chinese PLA General Hospital National Clinical Research Center for Geriatric Diseases Beijing China; ^3^ Department of Cardiology Chinese PLA No. 371 Hospital Henan China

**Keywords:** AKT, cardiac dysfunction, diabetic cardiomyopathy, FoxO1, Kir6.1

## Abstract

Previous studies have shown that the expression of inwardly rectifying potassium channel 6.1 (Kir6.1) in heart mitochondria is significantly reduced in type 1 diabetes. However, whether its expression and function are changed and what role it plays in type 2 diabetic cardiomyopathy (DCM) have not been reported. This study investigated the role and mechanism of Kir6.1 in DCM. We found that the cardiac function and the Kir6.1 expression in DCM mice were decreased. We generated mice overexpressing or lacking *Kir6.1* gene specifically in the heart. Kir6.1 overexpression improved cardiac dysfunction in DCM. Cardiac‐specific Kir6.1 knockout aggravated cardiac dysfunction. Kir6.1 regulated the phosphorylation of AKT and Foxo1 in DCM. We further found that Kir6.1 overexpression also improved cardiomyocyte dysfunction and up‐regulated the phosphorylation of AKT and FoxO1 in neonatal rat ventricular cardiomyocytes with insulin resistance. Furthermore, FoxO1 activation down‐regulated the expression of Kir6.1 and decreased the mitochondrial membrane potential (ΔΨm) in cardiomyocytes. FoxO1 inactivation up‐regulated the expression of Kir6.1 and increased the ΔΨm in cardiomyocytes. Chromatin immunoprecipitation assay demonstrated that the *Kir6.1* promoter region contains a functional FoxO1‐binding site. In conclusion, Kir6.1 improves cardiac dysfunction in DCM, probably through the AKT‐FoxO1 signalling pathway.

## 1 INTRODUCTION

Diabetic cardiomyopathy (DCM), characterized by structural, morphological, functional and metabolic abnormalities in the heart, severely impairs the health of diabetic patients and often occurs independently of myocardial ischaemia, congenital heart disease, hypertension and other cardiovascular diseases. Numerous molecular mechanisms have been proposed to contribute to the development of DCM, including altered myocardial insulin signalling, mitochondrial dysfunction, increased oxidative stress, autophagy and dysregulation of Ca^2+^ handling,^1^ which result in cardiomyocyte necrosis, cardiac remodelling and both diastolic and systolic dysfunction. Among these, altered myocardial insulin signalling may be the most common feature linking diabetes‐induced alterations to the development of cardiac dysfunction.^2^

Our previous studies have found that prolonged high‐fat diet (HFD) feeding of animal models impairs protein kinase B (AKT) activation and forkhead box protein O1 (FoxO1) transcription factor phosphorylation, resulting in persistent FoxO1 nuclear localization and activation.^3^, ^4^ Our recent study showed that persistently high insulin levels result in a significant decrease in the expression of phosphorylated AKT (p‐AKT) and FoxO1 (p‐FoxO1), mitochondrial membrane potential (ΔΨm) and cardiac function in *db*/*db* mice, which indicates the links between altered insulin signalling and mitochondria in DCM.^5^

ATP‐sensitive potassium channel (K_ATP_) plays an important protective role in the heart through various signalling pathways. K_ATP_ activation protects cardiomyocytes during heart failure, decreases ischaemia/reperfusion injury and reduces the occurrence of arrhythmias.^6^ K_ATP_ is composed of two types of subunits, inwardly rectifying potassium channels and sulphonylurea receptors, and its subunit composition is tissue specific.^7^, ^8^, ^9^ There is a K_ATP_ channel in the inner membrane of mitochondria (mitoK_ATP_),^10^, ^11^, ^12^ which the inwardly rectifying potassium channel 6.1 (Kir6.1) is a part of mitoK_ATP_ channels in cardiomyocytes.^6^, ^8^ A previous study has shown that the expression of Kir6.1 in heart mitochondria is significantly reduced in the mouse model of type 1 diabetes.^13^ However, it has not been reported whether its expression and function are changed and what role it plays in type 2 DCM.

Therefore, we investigated the role and mechanism of Kir6.1 in type 2 DCM. By overexpressing and knocking out Kir6.1 in the mouse heart, we investigated the effect of Kir6.1 on cardiac function and on the expression of the AKT‐FoxO1 signalling pathway in DCM. Furthermore, using primary ventricular cardiomyocyte cultures, we studied the effect of the AKT‐FoxO1 signalling pathway on the expression of Kir6.1 and on the function of cardiomyocyte mitochondria.

## 2 MATERIALS AND METHODS

### 2.1 Animal preparation and DCM model

Male pathogen‐free C57BL/6J mice at 5 weeks of age were supplied by the Experimental Animal Center of PLA General Hospital. The study protocols were approved by the Ethics Committee of Chinese PLA General Hospital. All the mice were treated in strict accordance with the NIH guidelines or the guidelines from Directive 2010/63/EU of the European Parliament on the protection of animals used for scientific purposes. DCM was induced using a HFD (D12492; Research Diets, New Brunswick, NJ, USA) together with an single intraperitoneal injection of streptozotocin (STZ; Sigma‐Aldrich, St. Louis, MO, USA).^14^ The mice were randomly divided into two groups: the DCM group was fed an HFD for 4 weeks, injected with STZ (100 μg/g of bodyweight) and then fed with HFD for another 12 weeks; the control group was fed a regular diet (D12450J, Research Diets, New Brunswick, NJ, USA) and injected with the same volume of vehicle (0.1 mol/L sodium citrate, Sigma‐Aldrich). Mice were housed five per cage, with free access to food and water. The HFD (5.21 kcal/g) consists of 60% calories from fat, 20% carbohydrate and 20% protein and the regular diet (3.82 kcal/g) contains 10% fat, 70% carbohydrate and 20% protein. The data regarding food intake and caloric intake for each experimental group are listed in Table S1. Plasma glucose levels were measured at the beginning and 12 weeks after the STZ injection by a Contour glucose meter (Roche, Basel, Switzerland). Mice with a fasting plasma glucose of over 13.89 mmol/L were considered diabetic (Figures S1 and S2). Mouse serum samples were analysed for Insulin using commercial enzyme‐linked immunosorbent assay (ELISA) kits (Ray Biotech, Norcross, GA, USA) according to the manufacturer's instructions. The data of insulin sensitivity test for all animals are provided in Figure S3. The mice were killed with an anaesthetic overdose of pentobarbital (100 mg/kg of bodyweight, Sigma‐Aldrich) injected intraperitoneally to obtain their samples.

### 2.2 Primary cardiomyocyte isolation and cell culture

Primary cultures of neonatal rat ventricular cardiomyocytes (NRVMs) were prepared from hearts of 1‐2‐day‐old Sprague Dawley rats, as previously described.^5^ NRVMs were cultured in Dulbecco's modified Eagle medium (DMEM) containing 10% foetal bovine serum for 48 hours.

### 2.3 Viral vector construction and transduction

A recombinant adeno‐associated virus serotype 9 containing Kir6.1 (AAV‐9) and a recombinant adenovirus encoding Kir6.1 (Ad‐Kir6.1) were packaged by Shanghai HanBio Company (Shanghai, China). The AAV‐9 capsid has previously been reported to show a modest preference for cardiac tissue in vivo.^15^ The mice were randomized into two groups and injected with the null control virus (AAV‐C, 2.70 × 10^11^ GC/mL, 100 μL per mouse) or AAV‐9 (3.97 × 10^11^ GC/mL, 100 μL per mouse) via the tail vein before being fed standard rodent chow or an HFD. For the in vitro experiments, after 48 hours of cell culture, the medium was changed with fresh DMEM containing serum and NRVMs were transfected by adding adenoviruses expressing green fluorescent protein (Ad‐C, Viral titre 1.58 × 10^10^ PFU/mL) or GFP‐fused Kir6.1 (Ad‐Kir6.1, Viral titre 1.58 × 10^10^ PFU/mL). The adenovirus dose is indicated as multiplicity of infection (MOI). After 8 hours of infection, the medium was changed to fresh DMEM containing serum for another 8 hours of culturing, and then, the cells were serum starved for 8 hours. Then, insulin (100 nmol/L, 24 hours; Sigma‐Aldrich) was used to induce insulin resistance. Cells in the control group were treated with 100 nmol/L insulin for 0.5 hours. More details are provided in Figures S4 and S5, Table S2.

### 2.4 Echocardiography

The mice were anaesthetized with 3% isoflurane and continuously monitored for heart rate, breathing and temperature. Transthoracic two‐dimensional M‐mode echocardiography was performed on anesthetized mice using Vevo770 (VisualSonics, Toronto, Canada) equipped with a 30‐MHz transducer. Left ventricular dimensions were measured according to the American Society for Echocardiography leading‐edge method from at least three consecutive cardiac cycles. The echocardiography parameters included cardiac output (CO), ratio of mitral valve E velocity to A velocity (MV E/A), ratio of mitral valve E velocity to E′ velocity (MV E/E′), interventricular septal thickness (IVST), left ventricular ejection fraction (LVEF), left ventricular fractional shortening (LVFS), left ventricular volume at systole/diastole (LV Vol; s/d), left ventricular posterior wall thickness at systole/diastole (LVPW; s/d) and left ventricular internal dimension at systole/diastole (LVID; s/d).

### 2.5 Brain natriuretic peptide measurement

Mouse serum samples and culture supernatants of cells were analysed for brain natriuretic peptide (BNP) using commercial enzyme‐linked immunosorbent assay (ELISA) kits (Ray Biotech, Norcross, GA, USA) according to the manufacturer's instructions.

### 2.6 Histological analysis

Hearts were fixed in 4% paraformaldehyde solution, embedded in paraffin and sectioned (5 μm thickness). After dehydration, sections were stained with haematoxylin and eosin (H&E) and then viewed under a microscope (Olympus, Tokyo, Japan). For quantification, cell area measurements were performed on five similar sections, and 100 nucleated cells were randomly selected to measure the mean cell area.

### 2.7 Apoptosis analysis

Hearts were fixed in 10% paraformaldehyde and embedded in paraffin. Paraffin‐embedded sections were incubated at 60°C for 15 minutes, dewaxed and rehydrated. Heart tissue sections (5‐μm thick) were used for apoptosis detection with a TUNEL assay kit (Roche), as previously described.^4^

### 2.8 Transmission electron microscopy

Hearts were fixed in 2.5% glutaraldehyde overnight, followed by osmication and uranyl acetate staining, dehydration in alcohol and embedding in epoxy resin (Solarbio Life Science, Beijing, China). Ultrathin sections were stained with uranyl acetate and lead citrate (Sigma‐Aldrich). The sections were viewed and imaged under a transmission electron microscope (Hitachi, Tokyo, Japan).

### 2.9 RNA isolation and quantitative real‐time PCR analysis

RNA from heart tissue or NRVMs was extracted with TRIzol reagent (Invitrogen, Carlsbad, CA, USA). cDNA synthesis was performed with a PrimeScript^TM^ RT regent Kit (Takara, Kyoto, Japan). Quantitative real‐time PCR (qRT‐PCR) was performed in duplicate in a total reaction volume of 25 μL using SYBR‐Green master mix (Takara) and conventional protocols. The primer sequences for qRT‐PCR are listed in Table S3. Expression was normalized to that of the housekeeping gene, *36β4*. Quantitative data was calculated using the comparative CT method.

### 2.10 Protein analysis by Western blotting

Proteins from tissues or cell cultures were extracted and resolved by SDS‐PAGE and transferred to nitrocellulose membranes for immunoblotting analysis, using specific antibodies. The primary antibodies as follows: Kir6.1 (1:200; rabbit polyclonal, ab251809, Abcam, Cambridge, MA, USA), p‑AKT (1:5,000; rabbit monoclonal, ab81283, Abcam), t‐AKT (1:10,000; rabbit monoclonal, ab179463, Abcam), p‑FoxO1 (1:300; rabbit polyclonal, ab131339, Abcam), t‑FoxO1 (1:300; rabbit polyclonal, ab39670, Abcam), GAPDH (1:2,000; rabbit monoclonal, ab181602, Abcam). The signal intensity was measured and analysed by Image J software, as previously described.^3^ The expression of specific proteins was normalized to the protein expression of GAPDH.

### 2.11 Oxygen consumption rate measurement

A Seahorse Bioscience XFe96 extracellular flux analyser was used to measure the oxygen consumption rate (OCR) in NRVMs using a previously reported protocol.^16^ NRVMs were plated at 3000 cells per well in XF media supplemented with pyruvate (1 mmol/L), glutamine (2 mmol/L) and glucose (10 mmol/L; Sigma‐Aldrich). Four independent OCR measurements were taken for each condition: baseline, and following the addition of oligomycin (1 μmol/L), FCCP (2 μmol/L), and antimycin A (0.5 μmol/L) plus rotenone (0.5 μmol/L; Agilent, Santa Clara, CA, USA). The protein concentration of NRVMs was determined for each well using a standard Bradford assay. Data were analysed by the Wave software and Report generator.

### 2.12 Generation of cardiac‐specific *Kir6.1*‐knockout mice

Kir6.1^lox/lox^ mice and αMHC‐MerCreMer mice (C57BL/6J background) were purchased from Guangzhou Cyagen Company (Guangzhou, China). Kir6.1^lox/lox^ mice were crossed with αMHC‐MerCreMer mice to generate cardiac‐specific *Kir6.1*‐knockout (Kir6.1^lox/lox^/MerCreMer, KO) mice. The Cre‐mediated excision of the Kir6.1 floxed‐allele in the heart was induced by daily intraperitoneal tamoxifen (20 mg/kg/d; Sigma‐Aldrich) injections for five consecutive days. Two days later, the KO&DCM group was fed an HFD for 4 weeks, injected with STZ (100 μg/g of bodyweight) and then fed with HFD for another 12 weeks; the control group was fed a regular diet and injected with the same volume of vehicle (0.1 mol/L sodium citrate). Littermate Kir6.1^wt/lox^/MerCreMer (K‐C) mice were used as controls.

### 2.13 Mitochondrial membrane potential measurement

After 48 hours of cell culture, NRVMs were divided into three groups: control, MK‐2206 dihydrochloride (5 μmol/L, 0.5 hours, Selleck Chemicals, Houston, TX, USA) plus insulin (100 nmol/L, 3 hours; MK‐2206&INS) and insulin (100 nmol/L, 3 hours; INS). Transitory insulin stimulation activates AKT and thereby inactivates FoxO1. MK‐2206 dihydrochloride (MK‐2206) is an AKT inhibitor, AKT inhibition activates FoxO1.^3^, ^4^ The ΔΨm of NRVMs was measured using the fluorescent dye, JC‑1 (Beyotime Biotechnology, Shanghai, China) as described previously.^5^ The images were analysed by Image Pro Plus 6.0 software. The ΔΨm was determined by calculating the ratio of red fluorescence to green fluorescence.

### 2.14 Chromatin immunoprecipitation assay

Chromatin immunoprecipitation assay was performed as described previously.^3^, ^17^ Heart chromatin was isolated from mice by immunoprecipitation with anti‐FoxO1 antibodies or control IgG. Occupancy of the FoxO1 site in the *Kir6.1* promoter was determined by PCR; control immunoprecipitation with nonrelevant IgG demonstrated the specificity of the assay. The primer sequences for PCR: 5′‐CCGTCCTGCTGGGTGTAAAT‐3′ and 5′‐ATATAGAGGGGTGGGAGGGC‐3′.

### 2.15 Statistical analysis

Statistical analysis was performed with SPSS 17.0 software. Data are expressed as the mean ± SEM (standard errors). Comparisons of parameters between two groups were performed with unpaired Student's *t*‐test. Comparisons of parameters among groups were determined by one‐way or two‐way ANOVA, followed by Tukey's post hoc test. *P* < .05 was considered statistically significant.

## 3 RESULTS

### 3.1 Cardiac and mitochondrial function are decreased in DCM mice and in insulin‐resistant NRVMs

**FIGURE 1:**
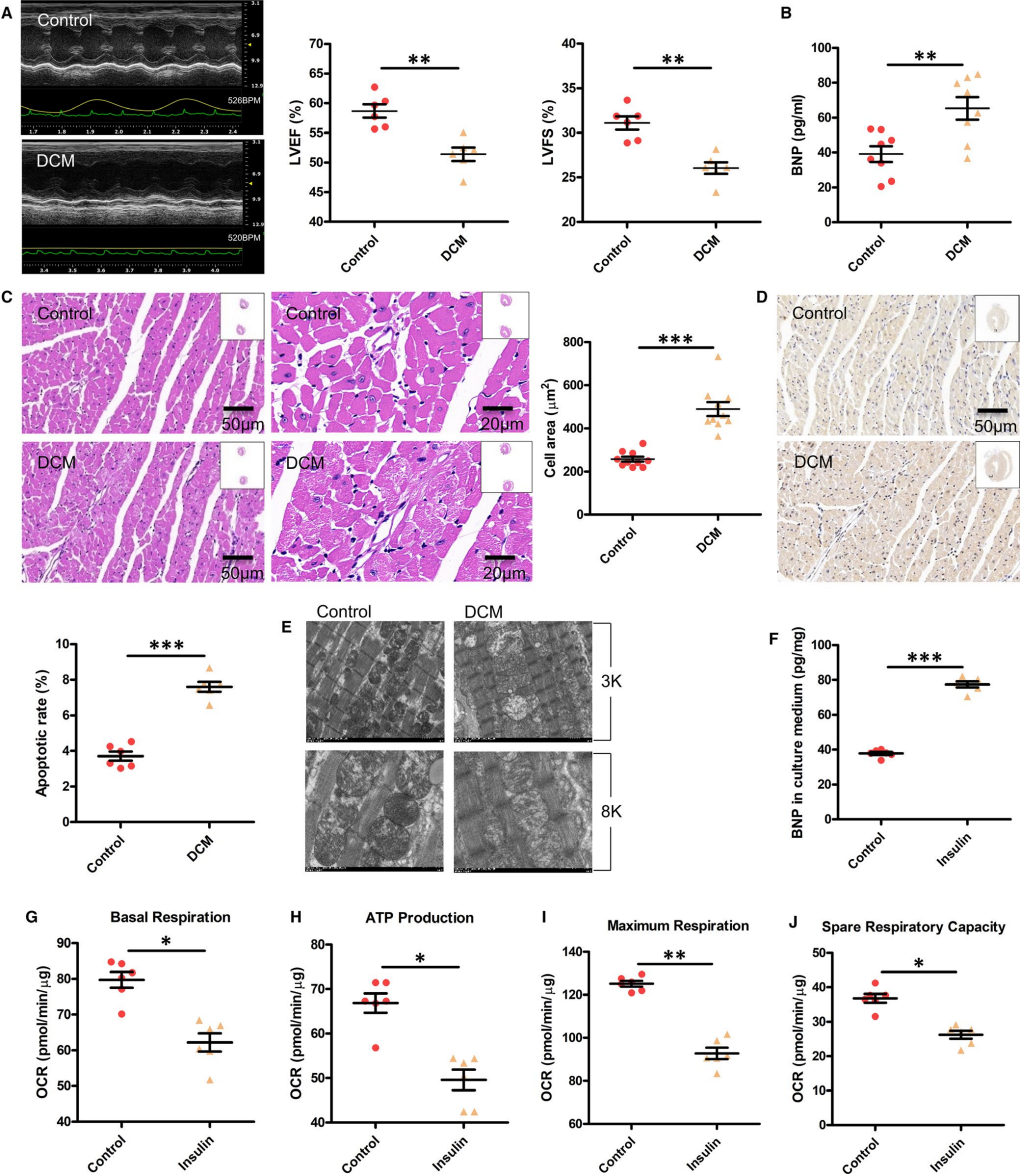
Cardiac and mitochondrial function are decreased in DCM mice and in insulin‐resistant NRVMs. A, The cardiac function of mice was determined by echocardiography. LVEF, left ventricular ejection fraction; LVFS, left ventricular fractional shortening. Values are expressed as the mean ± SEM. N = 6 mice in each group. *P*‐values from unpaired Student's *t*‐test: **P < .01 vs. control. B, Expression of brain natriuretic peptide (BNP) in the serum was analysed by ELISA. Values are expressed as the mean ± SEM. N = 8 mice. *P*‐values from unpaired Student's *t*‐test: **P < .01 vs. control. C, Cardiac histology. Representative transverse section from ten mice of left ventricle stained with haematoxylin and eosin (Scale bar = 50 μm/20 μm). For quantification, cell area measurements were performed on five similar sections (Scale bar = 50 μm), and 100 nucleated cells were randomly selected for measuring the mean cell area. Values are expressed as the mean ± SEM. N = 10 mice. *P*‐values from unpaired Student's *t*‐test: ***P < .001 vs. control. D, Apoptosis was detected by the TUNEL assay. Representative images from six animals for each group. The number of apoptotic cells in similar sections were counted and are shown as a percentage. N = 6 mice. *P*‐values from unpaired Student's *t*‐test: ***P < .001 vs. control. E, The ultrastructure of hearts was analysed by electron microscopy. Representative images from six mice of hearts with original magnification of ×3k (3000) and ×8k (8000) are shown. F, BNP expression in the culture medium was analysed by ELISA. Values are expressed as the mean ± SEM. N = 6 independent experiments in each group. *P*‐values from unpaired Student's *t*‐test: ***P < .001 vs. control. G‐J, Seahorse XFe96 Analyzer was used to test the mitochondrial function in NRVMs, including basal respiration, ATP production, maximal respiration and spare respiratory capacity. OCR, oxygen consumption rate. Values are expressed as the mean ± SEM. N = 6 independent experiments. *P*‐values from unpaired Student's *t*‐test: *P < .05 vs. control, **P < .01 vs. control

The cardiac function of DCM and control mice was analysed by echocardiography. Compared with the control mice, the DCM mice showed a significant reduction in CO, LVEF, LVFS and HW/BW, and an increase in LVPW; d, thereby exhibiting DCM (Table 1 and Figure 1A). ELISA demonstrated that the BNP protein expression was significantly increased in the heart of diabetic mice (Figure 1B). The myocardial structure was examined by H&E staining. Diabetic hearts displayed structural abnormalities, including abnormal cellular structures, the existence of foci with necrotic myocytes and increased cardiomyocyte areas (Figure 1C). The TUNEL assay was performed to examine apoptosis of cardiomyocytes. The proportion of apoptotic cells was remarkably increased in diabetic hearts compared with the controls (Figure 1D). We further examined the cardiac ultrastructure by transmission electron microscopy. Well‐organized morphology of sarcomeres, mitochondria and Z‐line was observed in the control hearts. However, diabetic hearts exhibited enlarged sarcomeres and abnormal changes in mitochondrial structure, including irregular arrangement, swelling and vacuolated and disrupted cristae (Figure 1E). These results indicated the successful establishment of the in vivo HFD&STZ‐induced DCM mouse model.

**TABLE 1:** Parameters of cardiac function as assessed by echocardiography

Variable	Overexpression				Knockout			
	Control	DCM	AVV‐9	AVV‐9&DCM	Control	DCM	KO	KO&DCM
BW (g)	31.86 ± 0.34	32.95 ± 0.72	25.32 ± 0.47	28.72 ± 0.79^#^	26.71 ± 0.37	29.78 ± 0.93	25.46 ± 0.92	30.26 ± 2.20
HW (mg)	182.68 ± 1.66	171.57 ± 5.91	131.90 ± 4.58	162.03 ± 9.87	152.09 ± 2.23	146.47 ± 7.14	141.85 ± 5.54	141.30 ± 15.53^#^
HW/BW (mg/g)	5.73 ± 0.04	5.21 ± 0.16*	5.21 ± 0.14	5.63 ± 0.23	5.69 ± 0.44	5.25 ± 0.09*	5.57 ± 0.10	4.63 ± 0.18^#^
HR (BPM)	541.53 ± 21.66	529 ± 31.33	537.24 ± 24.65	542.31 ± 19.21	545.34 ± 28.01	538.70 ± 20.30	548.82 ± 29.80	533.62 ± 30.89
LVEF (%)	58.68 ± 1.72	51.44 ± 1.74**	58.96 ± 2.85	55.71 ± 0.25^#^	58.19 ± 1.32	51.14 ± 1.31**	55.96 ± 0.54	43.12 ± 0.42^##^
LVFS (%)	30.89 ± 1.14	26.12 ± 1.02**	30.74 ± 1.97	28.87 ± 0.08^#^	31.96 ± 1.03	27.93 ± 1.16**	28.80 ± 0.40	21.06 ± 0.26^##^
LVID; s (mm)	3.06 ± 0.11	3.25 ± 0.16	2.61 ± 0.23	3.10 ± 0.04	2.96 ± 0.80	3.15 ± 0.84	2.87 ± 0.05	3.59 ± 0.18
LVID; d (mm)	4.42 ± 0.09	4.39 ± 0.17	3.75 ± 0.22	4.36 ± 0.02	4.26 ± 0.36	4.24 ± 0.23	4.03 ± 0.09	4.55 ± 0.21
LV Vol; s (μL)	36.82 ± 3.10	43.20 ± 4.97	25.89 ± 5.48	38.08 ± 1.09	34.81 ± 2.53	41.09 ± 5.14	31.34 ± 1.33	54.74 ± 6.40
LV Vol; d (μL)	88.69 ± 4.21	88.11 ± 7.85	61.03 ± 8.25	85.74 ± 0.94	85.91 ± 4.19	86.68 ± 8.65	71.29 ± 3.80	95.67 ± 1.05
LVPW; s (mm)	1.04 ± 0.06	1.11 ± 0.04	0.96 ± 0.05	0.92 ± 0.01^##^	1.02 ± 0.08	1.08 ± 0.06	1.01 ± 0.01	0.80 ± 0.06^###^
LVPW; d (mm)	0.69 ± 0.05	0.79 ± 0.04*	0.63 ± 0.04	0.68 ± 0.01^#^	0.68 ± 0.03	0.78 ± 0.01*	0.71 ± 0.02	0.57 ± 0.02^###^
IVST (mm)	0.66 ± 0.03	0.65 ± 0.02	0.60 ± 0.04	0.55 ± 0.02^#^	0.65 ± 0.05	0.64 ± 0.01	0.63 ± 0.03	0.57 ± 0.01^#^
CO (mL/min)	46.04 ± 0.70	31.81 ± 3.87*	40.04 ± 1.98	29.12 ± 4.98	44.04 ± 0.62	30.81 ± 3.25*	39.11 ± 4.71	15.40 ± 3.46^##^
MV E/A	2.08 ± 0.29	1.64 ± 0.19	1.91 ± 0.10	1.38 ± 0.01	2.00 ± 3.32	1.60 ± 0.13	1.57 ± 0.03	1.51 ± 0.12
MV E/E'	28.98 ± 2.34	31.64 ± 1.29	26.25 ± 0.84	25.61 ± 1.57^##^	28.87 ± 2.21	32.06 ± 1.15	32.05 ± 1.32	33.36 ± 0.12

Note

Values are presented as mean ± SEM N = 6 mice in each group.

Abbreviations: BW, bodyweight; HW, heart weight; LVEF, left ventricular ejection fraction; LVFS, left ventricular fractional shortening; LVID; s, left ventricular internal dimension at systole; LVID; d, left ventricular internal dimension at diastole; LV Vol; s, left ventricular volume at systole; LV Vol; d, left ventricular volume at diastole; LVPW; s, left ventricular posterior wall thickness at systole; LVPW; d, diastole; IVST, interventricular septal thickness; CO, cardiac output; MV E/A, ratio of mitral valve E velocity to A velocity; MV E/E′, ratio of mitral valve E velocity to E′ velocity.

*P*‐values from ANOVA: *P < .05 vs. control, **P < .01 vs. control, *^#^P* < .05 vs. DCM group, *^##^P* < .01 vs. DCM group, *^###^P* < .001 vs. DCM group.

Subsequently, NRVMs were treated with insulin to induce the cardiomyocyte model of insulin resistance. The level of BNP in the culture supernatant was increased in chronic insulin‐treated NRVMs (Figure 1F). We further measured the OCR in cardiomyocytes by a Seahorse XFe96 Analyzer. Chronic insulin stimulation significantly decreased the OCR, as manifested by basal respiration, ATP production, maximal respiration and spare respiratory capacity (Figure 1G‐J).

### 3.2 The expression of Kir6.1 is decreased in HFD&STZ‐induced type 2 diabetic mice and in chronic insulin‐resistant NRVMs

**FIGURE 2:**
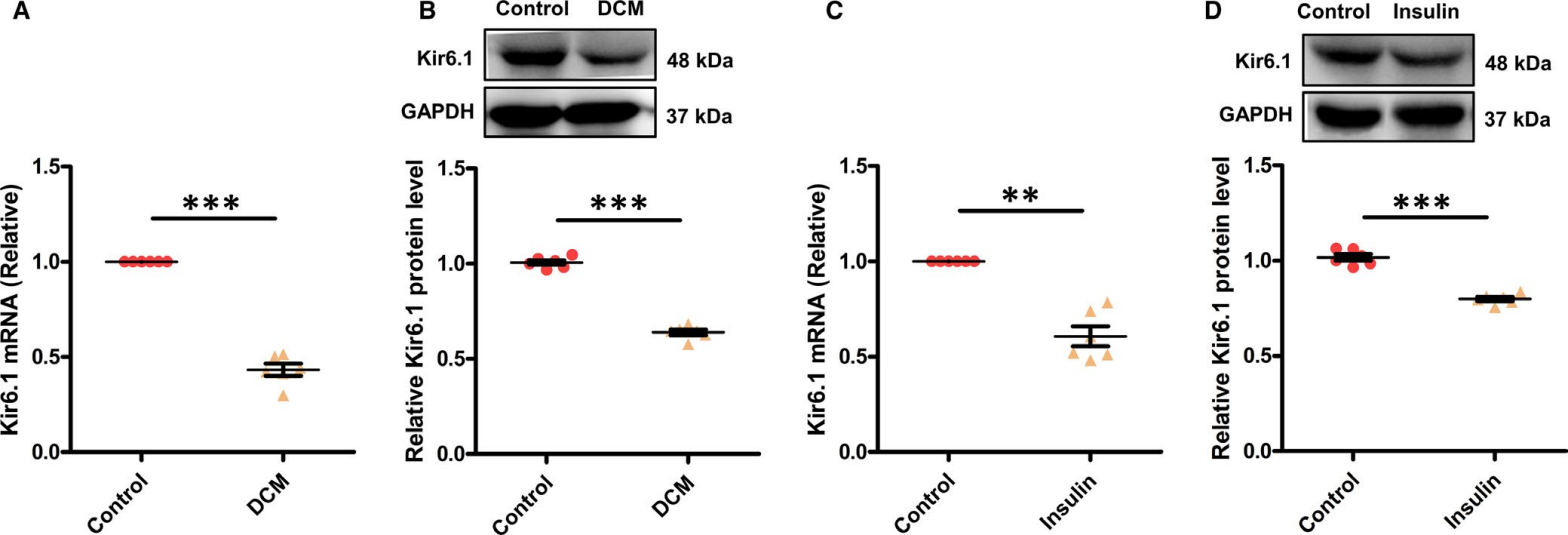
Kir6.1 in HFD&STZ‐induced type 2 diabetic mice and in chronic insulin‐resistant NRVMs determined by qRT‐PCR and Western blotting. A, B, Kir6.1 in mouse cardiac tissue. N = 6 mice in each group. C, D, Kir6.1 in NRVMs. N = 6 independent experiments in each group. Values are expressed as mean ± SEM *P*‐values from unpaired Student's *t*‐test: **P < .01 vs. control, ***P < .001 vs. control

qRT‐PCR analysis indicated that the *Kir6.1* mRNA expression was significantly decreased in DCM hearts (Figure 2A). Western blot analysis confirmed that Kir6.1 protein expression was reduced by 40% in DCM mice compared with the control mice (Figure 2B).

In accordance with the in vivo results, the mRNA and protein levels of Kir6.1 in NRVMs were obviously decreased after chronic insulin stimulation (Figure 2C and D). These results imply that Kir6.1 expression decreases with the decrease in cardiac function, suggesting it may play a role in DCM.

### 3.3 Kir6.1 overexpression improves cardiac dysfunction in DCM mice and in insulin‐resistant NRVMs

**FIGURE 3:**
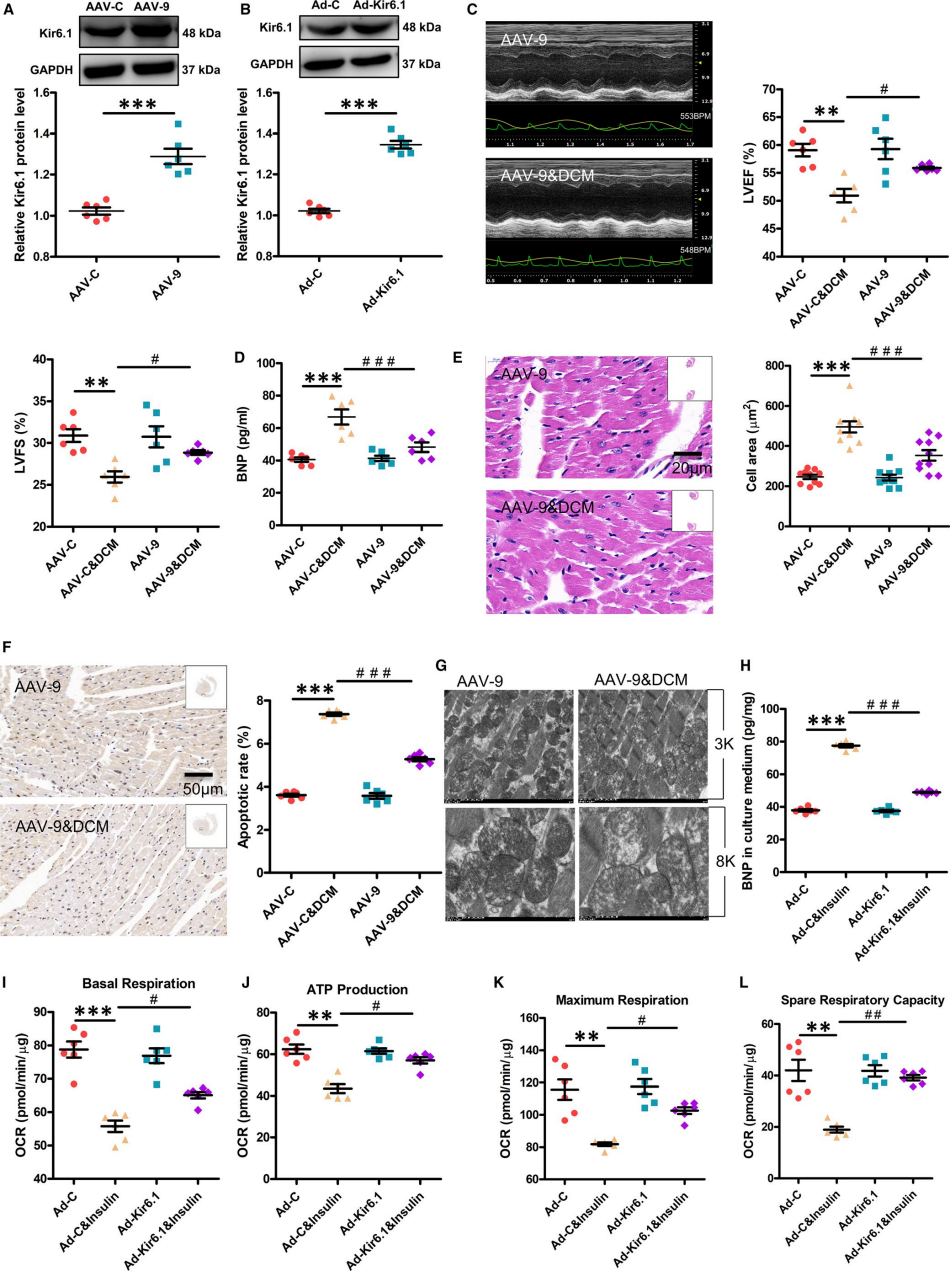
Kir6.1 overexpression improves cardiac dysfunction in DCM mice and in insulin‐resistant NRVMs. A, Kir6. 1 expression in mice was confirmed by Western blotting. Values are expressed as the mean ± SEM. N = 6 mice in each group. *P*‐values from unpaired Student's *t*‐test: ***P < .001 vs. control. B, Kir6.1 expression in NRVMs was confirmed by Western blotting. Values are expressed as the mean ± SEM. N = 6 independent experiments in each group. *P*‐values from unpaired Student's *t*‐test: ***P < .001 vs. control. C, Cardiac function in mice was determined by echocardiography. LVEF, left ventricular ejection fraction; LVFS, left ventricular fractional shortening. Values are expressed as the mean ± SEM. N = 6 mice. *P*‐values from ANOVA: **P < .01 vs. control, *^#^P* < .05 vs. DCM group. D, Brain natriuretic peptide (BNP) expression in the serum was analysed by ELISA. Values are expressed as the mean ± SEM. N = 6 mice. *P*‐values from ANOVA: ***P < .001 vs. control, *^###^P* < .001 vs. DCM group. E, Cardiac histology. Representative transverse section from ten mice of left ventricle stained with haematoxylin and eosin (Scale bar = 20 μm). For quantification, cell area measurements were performed on five similar sections (Scale bar = 50 μm), and 100 nucleated cells were randomly selected for measuring the mean cell area. Values are expressed as the mean ± SEM. N = 10 mice. *P*‐values from ANOVA: ***P < .001 vs. control, *^###^P* < .001 vs. DCM group. F, Apoptosis was detected by the TUNEL assay. Representative images from six animals for each group. The numbers of apoptotic cells in similar sections was counted and is shown as a percentage. N = 6 mice. *P*‐values from ANOVA: ***P < .001 vs. control, *^###^P* < .001 vs. DCM group. G, The ultrastructure of hearts was analysed by electron microscopy. Representative images from six mice of hearts with original magnification of ×3k (3000) and ×8k (8000) are shown. H, The BNP expression in the culture medium was analysed by ELISA. Values are expressed as the mean ± SEM. N = 6 independent experiments. *P*‐values from ANOVA: ***P < .001 vs. control, *^###^P* < .001 vs. Insulin group. I‐L, Seahorse XFe96 Analyzer was used to test the mitochondrial function in NRVMs, including basal respiration, ATP production, maximal respiration and spare respiratory capacity. OCR, oxygen consumption rate. Values are expressed as the mean ± SEM. N = 6 independent experiments. *P*‐values from ANOVA: **P < .01 vs. control, ***P < .001 vs. control, *^#^P* < .05 vs. Insulin group, *^##^P* < .01 vs. Insulin group

Kir6.1 expression was confirmed by Western blotting. The mice infected with AAV‐9 exhibited higher protein levels of Kir6.1 by about 1.3‐fold compared with the control (Figure 3A). To evaluate the efficacy of the *Kir6.1* gene transfection, NRVMs were transfected with Ad‐C at different MOIs (30, 50, 80 and 100). The best transfection efficiency was detected in the cells transfected with Ad‐C at an MOI of 80 (Figure S6). The NRVMs transfected with Ad‐Kir6.1 showed up‐regulated Kir6.1 protein expression compared with the cells transfected with the control adenovirus (Figure 3B).

The echocardiography data showed the protective effects of Kir6.1 on the HFD&STZ‐induced decrease in LVEF and LVFS (Figure 3C). Kir6.1 overexpression obviously suppressed the BNP protein level in the DCM mice (Figure 3D). It also alleviated HFD&STZ‐induced cardiomyocyte injury. The inhibitory effects of Kir6.1 on cardiac hypertrophy were further confirmed by the quantitative measurements of the cardiomyocyte area determined by H&E staining (Figure 3E). Furthermore, Kir6.1 overexpression significantly reduced diabetes‐induced myocardial cell apoptosis (Figure 3F) and rescued the myocardial morphology in DCM mice (Figure 3G).

Kir6.1 overexpression in chronic insulin‐stimulated NRVMs significantly decreased the level of BNP in the culture supernatant (Figure 3H). Furthermore, this overexpression attenuated the mitochondrial respiration dysfunction in chronic insulin‐stimulated NRVMs, including basal respiration, ATP production, maximal respiration and spare respiratory capacity (Figure 3I‐L).

### 3.4 Cardiac‐specific *Kir6.1* knockout aggravates cardiac dysfunction in diabetic mice

**FIGURE 4:**
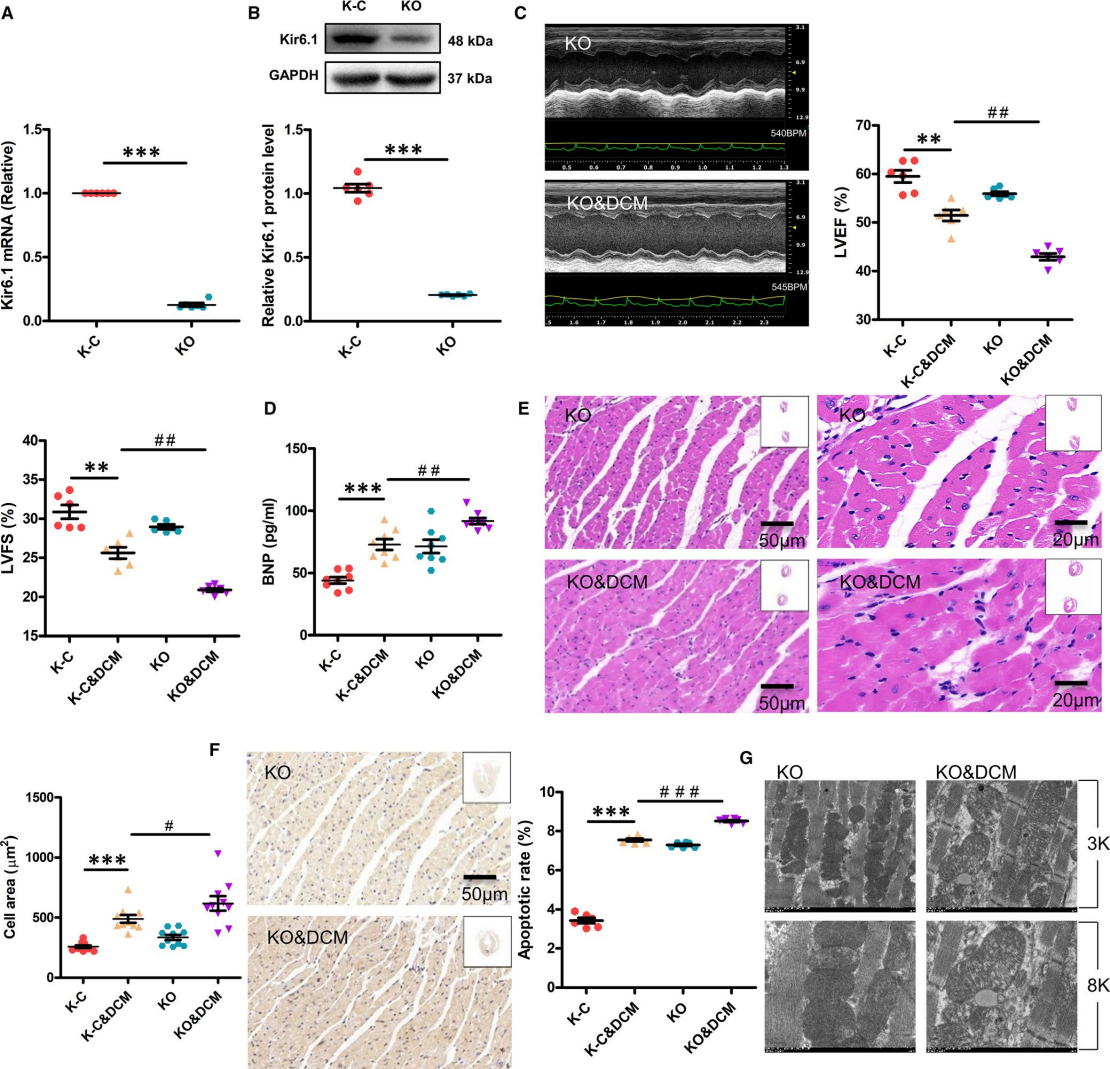
Cardiac‐specific *Kir6.1* knockout aggravates cardiac dysfunction in diabetic mice. A, The *Kir6.1* mRNA expression in the heart of mice was examined by qRT‐PCR. Values are expressed as the mean ± SEM. N = 6 mice in each group. *P*‐values from unpaired Student's *t*‐test: ***P < .001 vs. control. B, The Kir6.1 protein expression in the heart of mice was examined by Western blot analysis. Values are expressed as the mean ± SEM. N = 6 mice. *P*‐values from unpaired Student's *t*‐test: ***P < .001 vs. control. C, Mouse cardiac function was determined by echocardiography. LVEF, left ventricular ejection fraction; LVFS, left ventricular fractional shortening. Values are expressed as the mean ± SEM. N = 6 mice. *P*‐values from ANOVA: **P < .01 vs. control, *^##^P* < .01 vs. DCM group. D, Brain natriuretic peptide (BNP) expression in the serum was analysed by ELISA. Values are expressed as the mean ± SEM. N = 8 mice. *P*‐values from ANOVA: ***P < .001 vs. control, *^##^P* < .01 vs. DCM group. E, Cardiac histology. Representative transverse section from ten mice of left ventricle stained with haematoxylin and eosin (Scale bar = 50 μm/20 μm). For quantification, cell area measurements were performed on five similar sections (Scale bar = 50 μm), and 100 nucleated cells were randomly selected to measure the mean cell area. Values are expressed as the mean ± SEM. N = 10 mice. *P*‐values from ANOVA: ***P < .001 vs. control, *^#^P* < .05 vs. DCM group. F, Apoptosis was detected by the TUNEL assay. Representative images from six mice for each group. The number of apoptotic cells in similar sections was counted and is shown as a percentage. N = 6 mice. *P*‐values from ANOVA: ***P < .001 vs. control, *^###^P* < .001 vs. DCM group. G, The ultrastructure of hearts was analysed by electron microscopy. Representative heart images from six mice with original magnification of ×3k (3000) and ×8k (8000) are shown

We used cardiac‐specific *Kir6.1*‐knockout mice to further study the role of Kir6.1 in DCM. qRT‐PCR and Western blotting confirmed that the mRNA and protein levels of Kir6.1 in the heart of KO mice were significantly decreased compared with those in the control mice (Figure 4A and B).

Kir6.1 deficiency increased the deterioration in cardiac function induced by HFD&STZ, as manifested by the reduction in CO, HW/BW, IVST, LVEF and LVFS (Table 1 and Figure 4C). Kir6.1 deficiency in the heart resulted in higher BNP protein level in DCM mice compared with the control mice (Figure 4D). Cardiac‐specific Kir6.1 knockout also aggravated cardiac pathological changes in DCM mice, as demonstrated by the quantitative data of cardiomyocyte area determined by H&E staining (Figure 4E). Furthermore, the apoptosis rate in cardiac‐specific *Kir6.1*‐knockout DCM mice was significantly increased compared with that in DCM mice (Figure 4F). Moreover, Kir6.1 knockout exacerbated the myocardial morphology in DCM mice (Figure 4G).

### 3.5 Effect of Kir6.1 on the AKT‐FoxO1 signalling pathway in DCM

**FIGURE 5:**
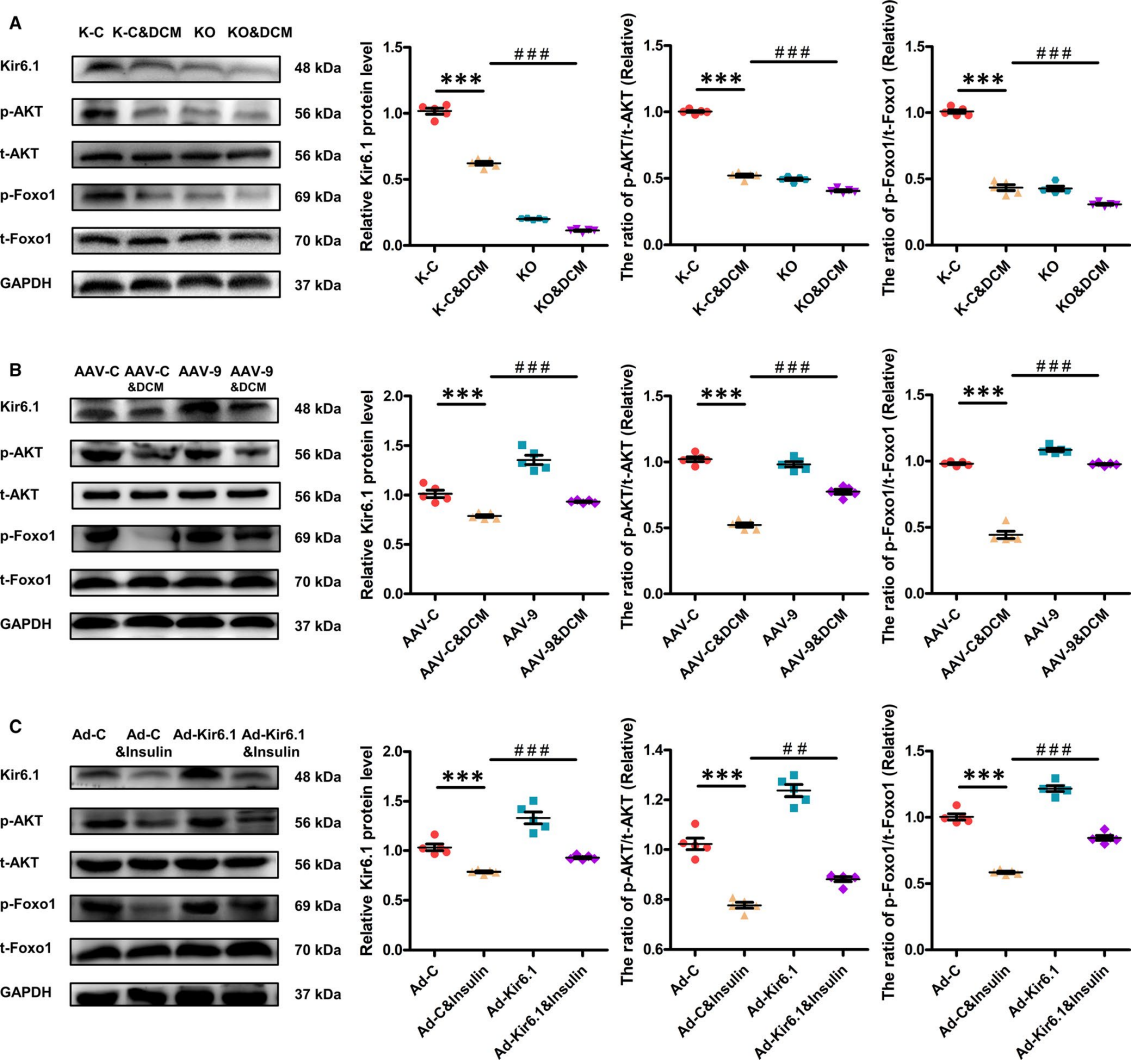
Effect of Kir6.1 on the AKT‐FoxO1 signalling pathway in DCM. A, Western blotting was performed to quantify the expression levels of Kir6.1, p‐AKT and p‐FoxO1 in the heart of mice. Values are expressed as the mean ± SEM. N = 5 mice in each group. *P*‐values from ANOVA: ***P < .001 vs. control, *^###^P* < .001 vs. DCM group. B, Western blotting was performed to quantify the expression levels of Kir6.1, p‐AKT and p‐FoxO1 in the heart of mice. Values are expressed as the mean ± SEM. N = 5 mice. *P*‐values from ANOVA: ***P < .001 vs. control, *^###^P* < .001 vs. DCM group. C, Western blotting was performed to quantify the expression levels of Kir6.1, p‐AKT and p‐FoxO1 in NRVMs. Values are expressed as the mean ± SEM. N = 5 independent experiments in each group. *P*‐values from ANOVA: ***P < .001 vs. control, *^##^P* < .01 vs. Insulin group, *^###^P* < .001 vs. Insulin group

To understand the mechanism by which Kir6.1 overexpression reduces cardiac dysfunction in DCM, the changes in the AKT‑FoxO1 signalling pathway were investigated in vivo and in vitro. The expression of p‐AKT and p‐FoxO1 was markedly down‐regulated in the DCM group and Insulin group. Kir6.1 knockout further decreased the phosphorylation of AKT and FoxO1 in DCM mice (Figure 5A). However, Kir6.1 overexpression increased the levels of p‐AKT and p‐FoxO1 in DCM mice (Figure 5B). Consistent with the in vivo results, the levels of p‐AKT and p‐FoxO1 were up‐regulated in the Ad‐Kir6.1&Insulin‐treated NRVMs (Figure 5C). These findings demonstrated that Kir6.1 overexpression attenuates cardiac dysfunction in DCM, probably through the AKT‐FoxO1 signalling pathway.

### 3.6 Effect of the AKT‐FoxO1 signalling pathway on the expression of Kir6.1 and the function of mitochondria in cardiomyocytes

**FIGURE 6:**
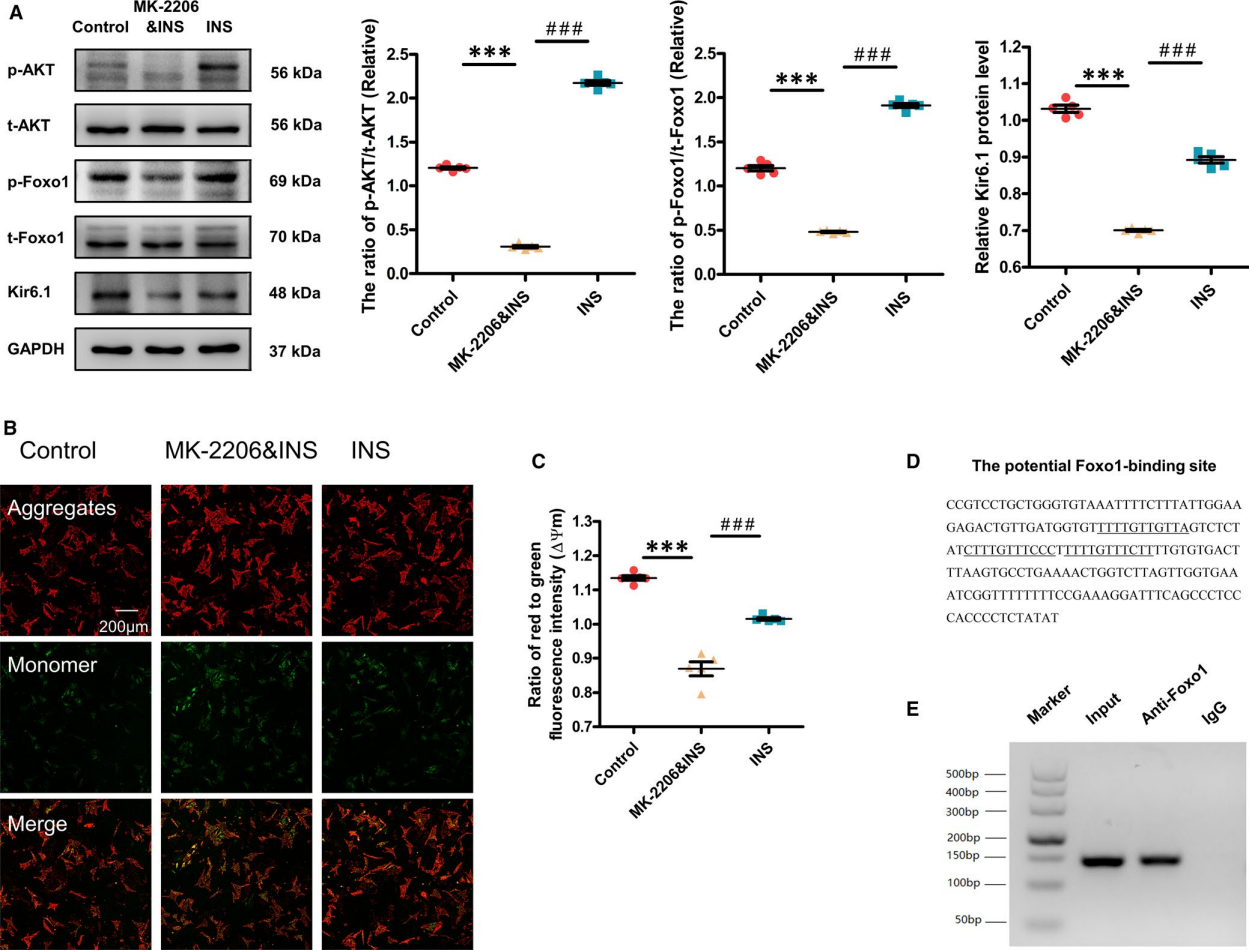
Effect of the AKT‐FoxO1 signalling pathway in regulating Kir6.1 expression and mitochondrial function in cardiomyocytes. A, Western blotting was performed to quantify the expression levels of Kir6.1, p‐AKT and p‐FoxO1 in cardiomyocytes. Values are expressed as the mean ± SEM. N = 5 independent experiments in each group. *P*‐values from ANOVA: ***P < .001 vs. control, *^###^P* < .001 vs. MK‐2206&INS group. B, Determination of ΔΨm in the three groups of cardiomyocytes by the fluorescent dye, JC‑1 (Scale bar = 200 μm). C, Comparison of the ΔΨm among the three groups of cardiomyocytes. For quantification, 20 cells were randomly selected to calculate the ΔΨm levels by comparing red fluorescent intensity to green fluorescent intensity. Values are expressed as the mean ± SEM. N = 5 independent experiments. *P*‐values from ANOVA: ***P < .001 vs. control, *^###^P* < .001 vs. MK‐2206&INS group. D, Potential FoxO1‐binding sites are located 2.0 kb upstream of the *Kir6.1* gene's transcription initiation site. E, Binding of endogenous FoxO1 from the hearts of mice to the potential FoxO1‐binding site was confirmed by the chromatin immunoprecipitation assay. Representative results from three independent experiments are shown

We next examined the role of the AKT‐FoxO1 signalling pathway in regulating Kir6.1 expression and mitochondrial function in cardiomyocytes. Transitory insulin stimulation activates AKT and thereby inactivates FoxO1. MK‐2206 is an AKT inhibitor, AKT inhibition activates FoxO1. The expression of p‐AKT, p‐FoxO1 and Kir6.1 was markedly decreased in the MK‐2206&INS group. In contrast, the expression of p‐AKT, p‐FoxO1 and Kir6.1 in the INS group was increased compared with the MK‐2206&INS group (Figure 6A).

In the MK‐2206&INS group, the ΔΨm was lower than that in the control group. However, in the INS group it was higher than that in the MK‐2206&INS group (Figure 6B and C).

Finally, we examined whether the promoter region of *Kir6.1* has a consensus FoxO1‐binding site. A region of 2.0 kb in the mouse *Kir6.1* promoter was analysed with the Jaspar genome database and three consecutive copies of the conserved FoxO1‐binding sequence were identified (Figure 6D). To determine whether FoxO1 interacts with this promoter region, chromatin immunoprecipitation experiments were performed, which indicated that endogenous FoxO1 interacted with the consensus DNA sequence in the *Kir6.1* promoter region (Figure 6E).

## 4 DISCUSSION

In this study, we investigated the role and mechanism of Kir6.1 in DCM. We found that the cardiac function and Kir6.1 expression were decreased in DCM mice. Kir6.1 overexpression improved cardiac dysfunction and up‐regulated the phosphorylation of AKT and FoxO1 in DCM, both in vivo and in vitro. In contrast, cardiac‐specific Kir6.1 knockout aggravated the cardiac dysfunction and down‐regulated the phosphorylation of AKT and FoxO1 in DCM mice. Activation of FoxO1 down‐regulated the expression of Kir6.1 and decreased the ΔΨm in cardiomyocytes. In contrast, inactivation of FoxO1 up‐regulated the expression of Kir6.1 and increased the ΔΨm in cardiomyocytes. Furthermore, FoxO1 was shown to interact with the promoter region of *Kir6.1* for transcription activation.

Diabetic cardiomyopathy is becoming a well‐known clinical phenomenon. The metabolic milieu associated with diabetes, such as hyperglycaemia and hyperinsulinaemia, alters multiple molecular pathways within the cardiomyocyte, thereby impairing cardiac contractility and promoting myocyte dysfunction, injury and cell death.^1^ Systolic and diastolic dysfunction can be consistently reproduced in a variety of rodent models of diabetes. Echocardiography is a standard modality for diagnosing DCM. There are three myocardial signals in DCM: left ventricular (LV) diastolic dysfunction, abnormal LV systolic function and changes in LV geometry.^18^ Alteration in the BNP level suggests myocardial structural and functional dysfunction. Elevated BNP levels showed a positive correlation with LV dysfunction in DCM.^19^ Cardiomyocyte hypertrophy is a common structural hallmark in patients with DCM.^20^, ^21^ A relatively oxygen‐poor environment induced by hypertrophy accelerates cardiomyocyte apoptosis. Cardiac function is energetically demanding and thus reliant on efficient well‐coupled mitochondria to generate adenosine triphosphate (ATP). Extensive experimental results demonstrated that cardiomyocytes from animal models of type 1 and 2 diabetes had altered mitochondrial morphology and mitochondrial dysfunction.^22^ Chronic insulin stimulation degrades insulin receptor substrate 1 and 2 (IRS1 and IRS2) protein and causes insulin resistance in vitro. In this study, NRVMs were treated with 100 nmol/L insulin for 24 hours to induce the cardiomyocyte model of insulin resistance, as previously described.^3^, ^4^, ^5^ A study has shown that short‐term or long terms exposure to insulin (from 10^−11^ to 10^−7^ M) has different effects on the phosphoinositide 3‐kinase (PI3K)/AKT/NO‐dependent insulin signalling pathway in human umbilical vein endothelial cells. Insulin increases the active phosphorylated forms of AKT and eNOS in the cells treated with insulin in short‐term (30 minutes). But the effects are attenuated in the cells treated with insulin in long‐term (24 hours to 3 days).^23^, ^24^ In human myoblasts, treatment with 1430 pmol/L insulin for 3 weeks induces an up‐regulation of insulin receptor (IR), IRS1 and PI3K activity.^25^ Canine cardiac myoblasts treated with insulin (10^−8^ to 10^−7^ mol/L) for 24 hours increase the levels of phosphorylated IRS1.^26^

A recent study has confirmed the existence of mitoK_ATP_ encoded by the CCDC51 gene.^10^ K_ATP_ channels, a hetero‐octamers, is consisting of four pore‐forming Kir6.x (Kir6.1 or Kir6.2) subunits and four regulatory sulphonylurea receptor (SUR1 or SUR2) subunits.^27^ However, the composition of mitoK_ATP_ channels is still unclear. Kir6.1 protein is present in both the plasma membrane and the mitochondrial membrane. Kir6.1 is prominently expressed in vascular smooth muscle, skeletal muscle and glial cells.^27^ Some literature suggests that Kir6.1 may be a functionally important part of mitoK_ATP_ channels in native cardiomyocytes.^8^, ^28^ Alteration in mitochondrial function has been linked to cardiovascular diseases including DCM.^29^, ^30^ Furthermore, some studies have shown the cardioprotective roles of mitoK_ATP_.^31^ In our study, the expression of Kir6.1 was decreased in the mouse model of type 2 DCM. In accordance with the in vivo results, it was decreased in a cardiomyocyte model of insulin resistance, which was also consistent with a previous study.^13^ The data indicated that Kir6.1 may play a role in DCM. Therefore, transgenic mice overexpressing Kir6.1 or lacking Kir6.1 specifically in the heart were used to study the role of Kir6.1 in DCM. However, the transduction was systemic and not focused to the heart. Therefore, we cannot exclude some effect on peripheral vascular resistances. Previous studies have shown that AAV‐9 leads to preferential cardiac transduction in vivo.^15^, ^32^ Moreover, cardiomyocytes can be efficiently transfected by adenoviruses. Our data showed that Kir6.1 expression was overexpressed in vivo and in vitro after AAV‐9 or adenoviral gene transfer, respectively. The ability to control the tissue specificity of gene knockout in the rodent using the Cre‐loxP technology has profoundly advanced rodent genetics and the ability to examine single gene functions in vivo.^33^ We used the Cre‐loxP technology to modify gene expression in our mouse model. The expression of Kir6.1 in the heart was significantly decreased after intraperitoneal tamoxifen injection, indicating that the cardiac‐specific *Kir6.1*‐knockout mouse model was successfully established.

In this study, we found that the cardiac function in DCM mice was decreased, including systolic dysfunction, increase in BNP, cardiomyocyte hypertrophy and apoptosis, and abnormal changes in mitochondrial structure in vivo. Additionally, we found increased BNP levels and reduction in the OCR in vitro. DCM and its associated mitochondrial dysfunction have been observed in *ob*/*ob*, *db*/*db* and HFD‐fed mice.^34^, ^35^ Furthermore, cardiac tissue from Akita mice displayed swollen mitochondria, lacking a well‐defined cristae structure along with decreased states 3 and 4 respiration and ATP synthesis.^36^ Our data agree with many previous studies on cardiac dysfunction in rodent models of DCM.^37^, ^38^, ^39^, ^40^, ^41^ However, in the current study, Kir6.1 overexpression reduced cardiac dysfunction in diabetic mice and dysfunction of cardiomyocytes with insulin resistance, whereas cardiac‐specific Kir6.1 knockout aggravated cardiac dysfunction in diabetic mice. Thus, our findings suggest that Kir6.1 overexpression attenuates cardiac dysfunction in DCM.

Cardiac insulin signalling mediates cellular homeostasis by controlling substrate use, protein synthesis, autophagy and cell survival.^42^ Physiologically, binding of insulin to IR activates IRS1 and IRS2 and the downstream PI3K‐AKT pathways. AKT is required for cardiac growth, metabolism and survival, and its targets include p70S6K (protein synthesis), Glut4 (glucose transport) and FoxO1 (gene expression).^43^ Briefly, insulin exerts its function through AKT activation, which in turn phosphorylates FoxO1. In cardiomyocytes, FoxO1 is involved in the control of many important properties such as cell growth, metabolic adaptation, cell apoptosis, autophagy and resistance to oxidative stress.^44^, ^45^ Impaired glucose uptake in the diabetic heart is often linked with reduced expression or activity of the downstream intermediates in the insulin signalling pathway. In this study, the levels of p‐AKT and p‐FoxO1 were markedly down‐regulated in DCM. Decreased cardiac basal and insulin‐stimulated phosphorylation of AKT and FoxO1 is evident in diabetic mouse models.^46^ In our previous studies, prolonged HFD feeding of mouse models impaired AKT activation and FoxO1 phosphorylation, which resulted in persistent FoxO1 nuclear localization and activation,^3^, ^4^ consequently leading to cardiac dysfunction. Furthermore, our recent study showed a reduction in the expression of p‐AKT and p‐FoxO1 and in cardiac function in *db*/*db* mice.^5^ K_ATP_ plays a key protective role in the heart through various signalling pathways. Specifically, genetic manipulation of cardiomyocyte insulin signalling intermediates has demonstrated that partial cardiac function rescue was achieved by up‐regulation of the insulin signalling pathway in diabetic hearts.^47^ Similarly, a previous study has reported that the cardioprotective effect of K_ATP_ occurs at least partially by regulating the AKT‐FoxO1 signalling pathway, which in turn influences the expression of PGC‐1α and its downstream target genes.^48^ Our recent study also showed that opening of mitoK_ATP_ increased the phosphorylation of AKT and FoxO1, but the effects of this opening were blocked by the specific AKT inhibitor, MK‐2206.^5^ In our current study, Kir6.1 knockout further suppressed the phosphorylation of AKT and FoxO1 in DCM mice and increased cardiac dysfunction. On the contrary, Kir6.1 overexpression up‐regulated the phosphorylation of AKT and FoxO1 in DCM models and improved cardiac dysfunction both in vivo and in vitro. The above data indicate that Kir6.1 overexpression attenuates cardiac dysfunction in DCM, probably through the AKT‐FoxO1 signalling pathway.

FoxO1 and its downstream targets play a key role in mitochondrial biogenesis.^49^, ^50^, ^51^ Transient insulin stimulation activates the PI3K‐AKT signalling pathway and suppresses FoxO1 activation. Inactivation of AKT through an AKT‐specific inhibitor activated FoxO1. Activation of FoxO1 results in haem deficiency, limiting mitochondrial cofactor biosynthesis and ATP production.^3^, ^4^, ^52^ The stability of ΔΨm is important for energy conversion. A decrease in the ΔΨm affects energy conversion, leading to cell dysfunction.^53^ In our study, the AKT‐specific inhibitor, MK‐2206, prevented endogenous AKT activation in transient insulin stimulation, resulting in FoxO1 activation, decreased Kir6.1 expression and reduced ΔΨm. However, FoxO1 inactivation up‐regulated Kir6.1 expression and increased ΔΨm in the NRVMs stimulated by transient insulin. FoxO1 promotes loss of mitochondria by activating the gene expression of haemeoxygenase‐1, an enzyme that catalyses haem degradation. Haem is an essential component of mitochondrial complexes III and IV.^3^, ^52^ Chromatin immunoprecipitation assay demonstrated that the *Kir6.1* promoter region contains a functional FoxO1‐binding site. FoxO1 interacts with the promoter region of Kir6.1 for transcriptional activation. Our results indicate that the interaction between the AKT‐FoxO1 signalling pathway and Kir6.1 may play a key role in the pathogenesis of DCM.

Heart failure is the main cause of death in patients with type 2 diabetes, but the molecular mechanism of the link between diabetes mellitus and heart failure is not clear. Insulin resistance is a sign of type 2 diabetes. IRS1 and IRS2 are the main insulin signalling components that regulate cell metabolism and survival. Our previous studies have shown that IRS1 and IRS2 play important roles in controlling cardiac function, metabolism and homeostasis. And inhibition of cardiac IRS1 and IRS2 may be a fundamental mechanism for inducing heart failure.^4^ A previous study has demonstrated that failing heart with coronary patency shows insulin resistance, glycogen deposition and asymmetrical myocardial hibernation due to microcirculatory dysfunction.^54^ And diabetic cardiomyopathy is characterized by insulin resistance, chronic myocardial ischaemia and features of myocardial stunning/hibernation. Furthermore, another study showed that higher basal glycogen deposition was detected in *Kir6*.*2* knockout heart.^55^ Our previous study suggests that Foxo1 plays an important role in promoting diabetic cardiomyopathy and controlling β‐MHC expression in the development of cardiac dysfunction.^3^ The interaction between the AKT‐FoxO1 signalling pathway and Kir6.1 may therefore play a potential myocardial metabolic role in the onset of heart failure in light of the abovementioned study.

In this study, the isoflurane MAC (%) is too high and may seriously affect the systolic function without cardiac injury. NRVMs were treated with insulin to induce the cardiomyocyte model of insulin resistance, which is not so representative as cardiomyocytes isolated from the mice model of DCM, because of the difference phenotype of neonatal cardiomyocytes from adult ones. We also did not investigate the detailed molecular mechanism of Kir6.1’s action on the AKT‐FoxO1 signalling pathway, which is another limitation of this study. Our future study will focus on the molecular mechanisms of Kir6.1’s interaction with the AKT‐FoxO1 signalling pathway in DCM.

In conclusion, our results provided in vivo and in vitro evidence that Kir6.1 improves cardiac dysfunction in DCM, probably through the AKT‐FoxO1 signalling pathway. Moreover, the crosstalk between Kir6.1 and the AKT‐FoxO1 signalling pathway may provide new strategies for reversing the defective signalling in DCM.

## CONFLICT OF INTEREST

The authors declare that there are no conflict of interests.

## AUTHOR CONTRIBUTION

**Jinxin Wang:** Conceptualization (equal); Data curation (equal); Formal analysis (equal); Investigation (lead); Methodology (equal); Resources (equal); Software (equal); Writing‐original draft (lead). **Jing Bai:** Data curation (equal); Investigation (equal); Methodology (equal). **Peng Duan:** Data curation (equal); Investigation (equal); Methodology (equal). **Hao Wang:** Formal analysis (equal); Software (equal); Supervision (equal). **Yang Li:** Formal analysis (equal); Software (equal); Supervision (equal). **Qinglei Zhu:** Conceptualization (lead); Funding acquisition (lead); Project administration (lead); Writing‐review & editing (lead).

## Supporting information

Fig S1

Fig S2

Fig S3

Fig S4

Fig S5

Fig S6

Table S1

Table S2

Table S3

## Data Availability

The data sets used and/or analysed during the current study are available from the corresponding author on reasonable request.
