# Supplementary figures and images for: Kir6.1 improves cardiac dysfunction in diabetic cardiomyopathy via the AKT‐FoxO1 signalling pathway
Source: J Cell Mol Med. 2021 Feb 6;25(8):3935–49. doi: 10.1111/jcmm.16346 (PMC8051713; doi:10.1111/jcmm.16346)

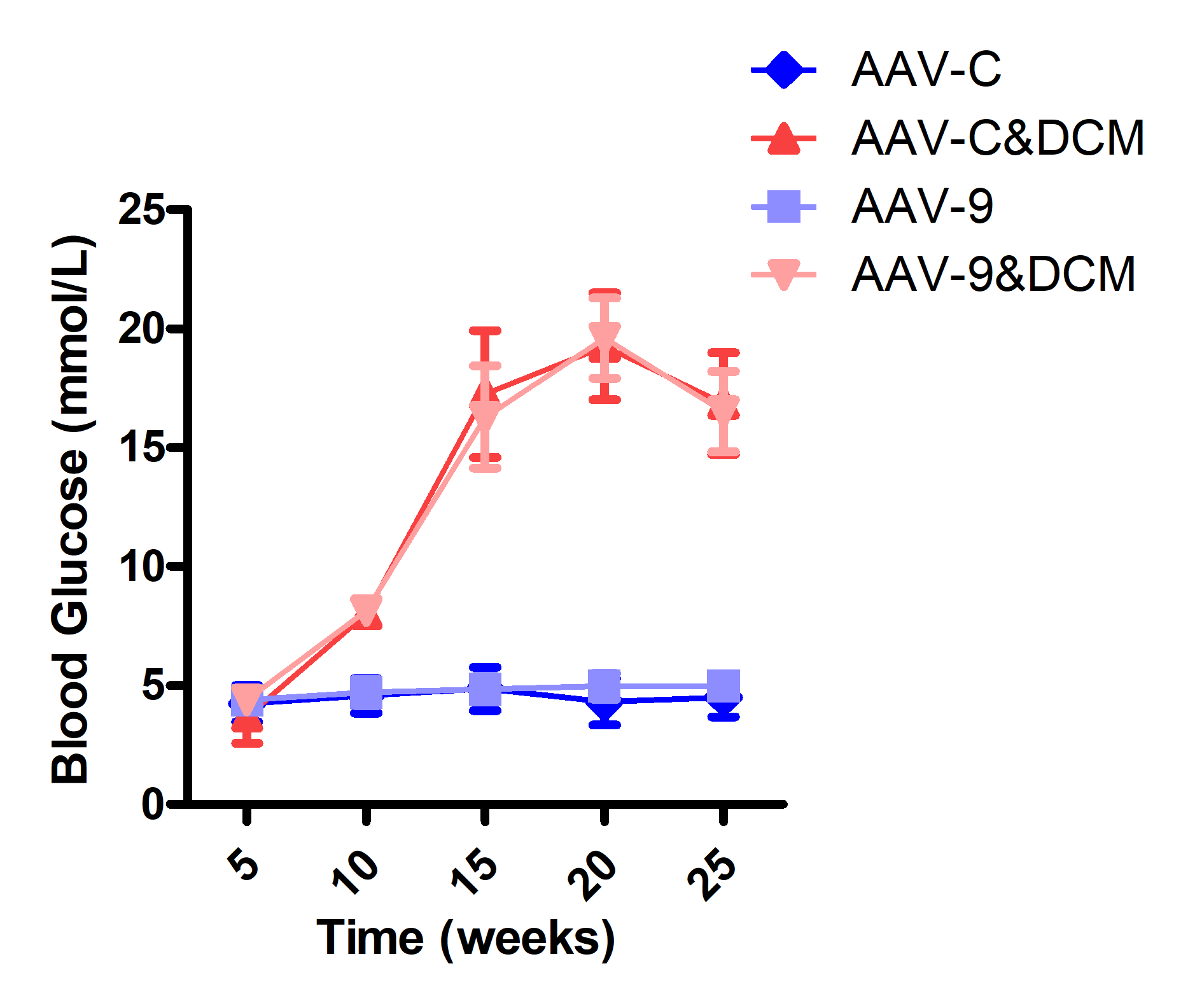

Supplement: Supplementary file 1 — Fig S1 [file JCMM-25-3935-s004.tif]

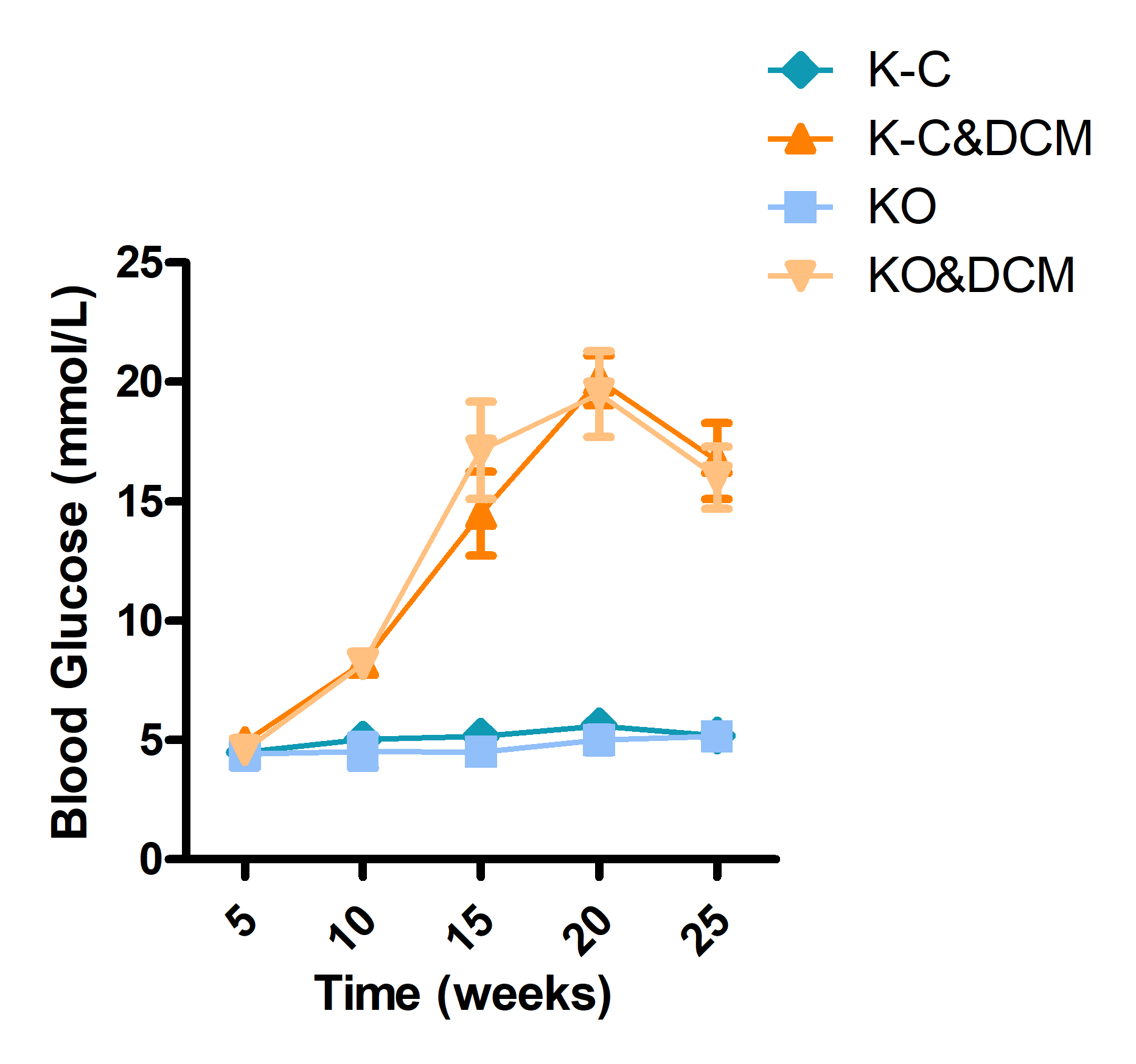

Supplement: Supplementary file 2 — Fig S2 [file JCMM-25-3935-s009.tif]

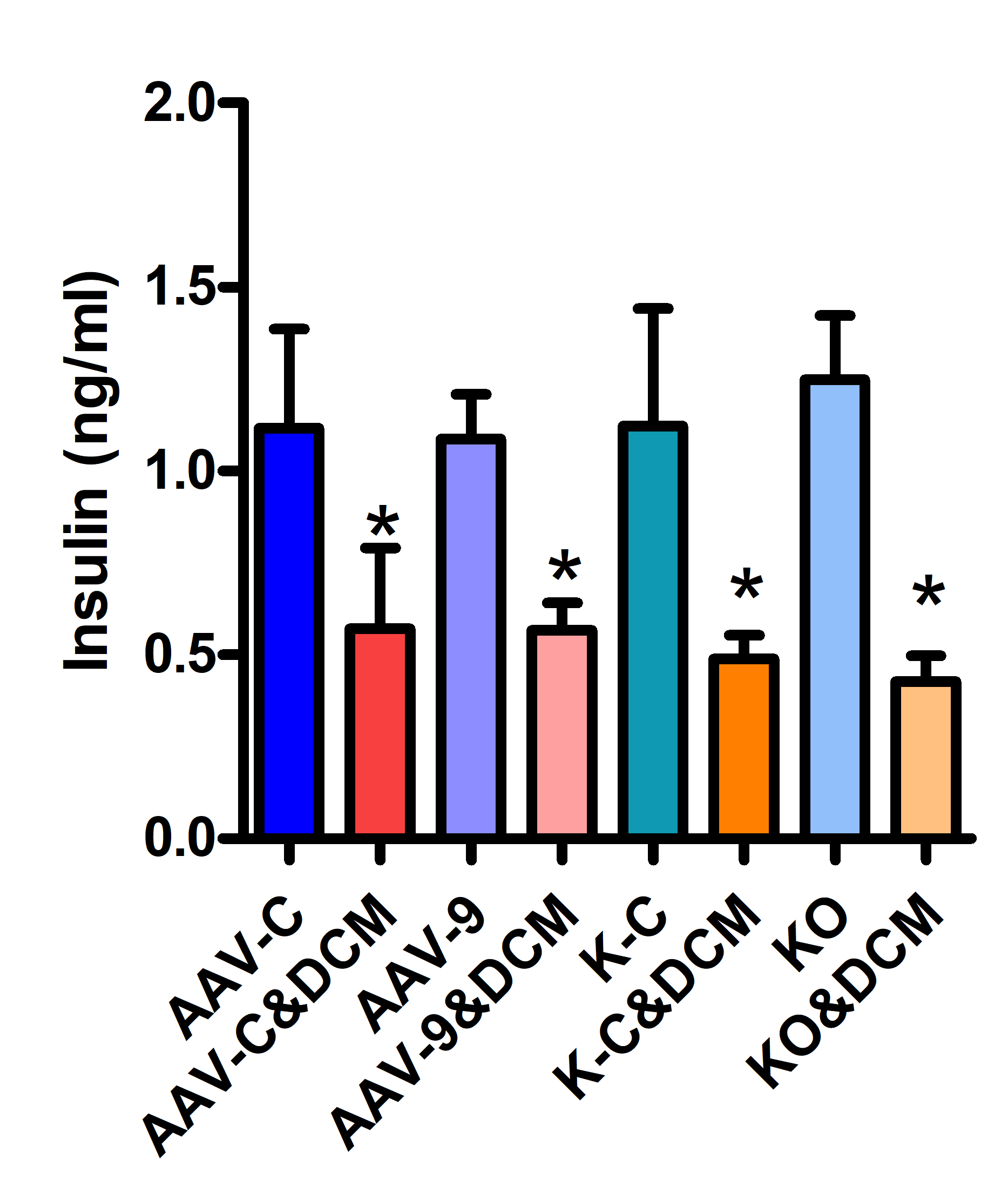

Supplement: Supplementary file 3 — Fig S3 [file JCMM-25-3935-s005.tif]

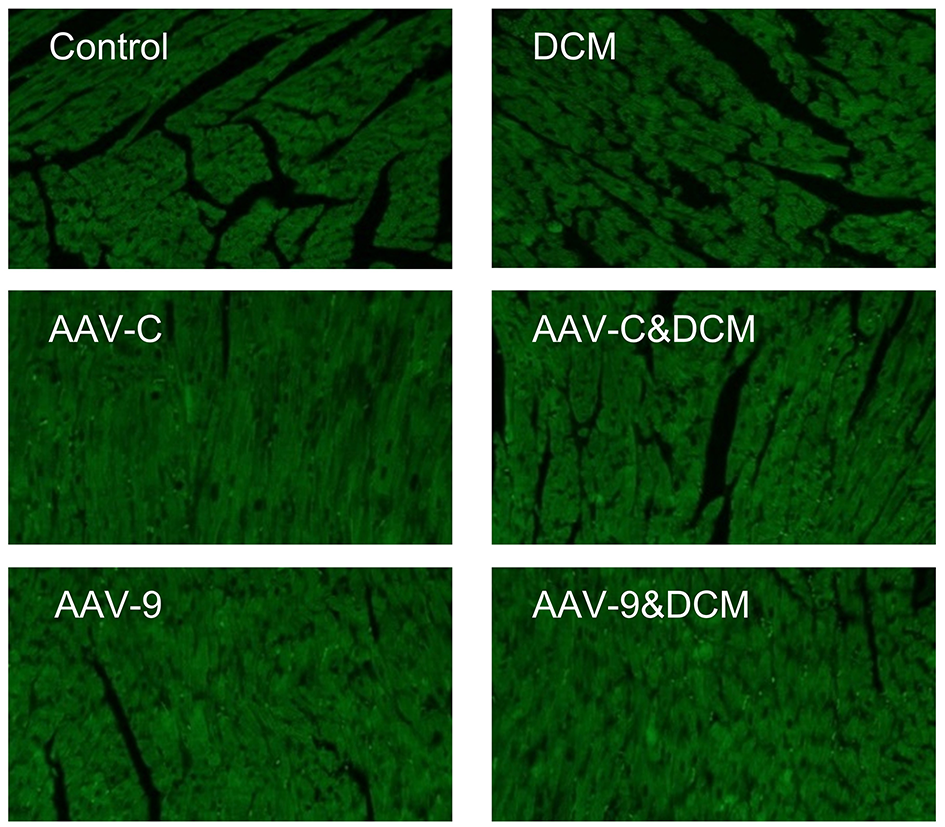

Supplement: Supplementary file 4 — Fig S4 [file JCMM-25-3935-s002.tif]

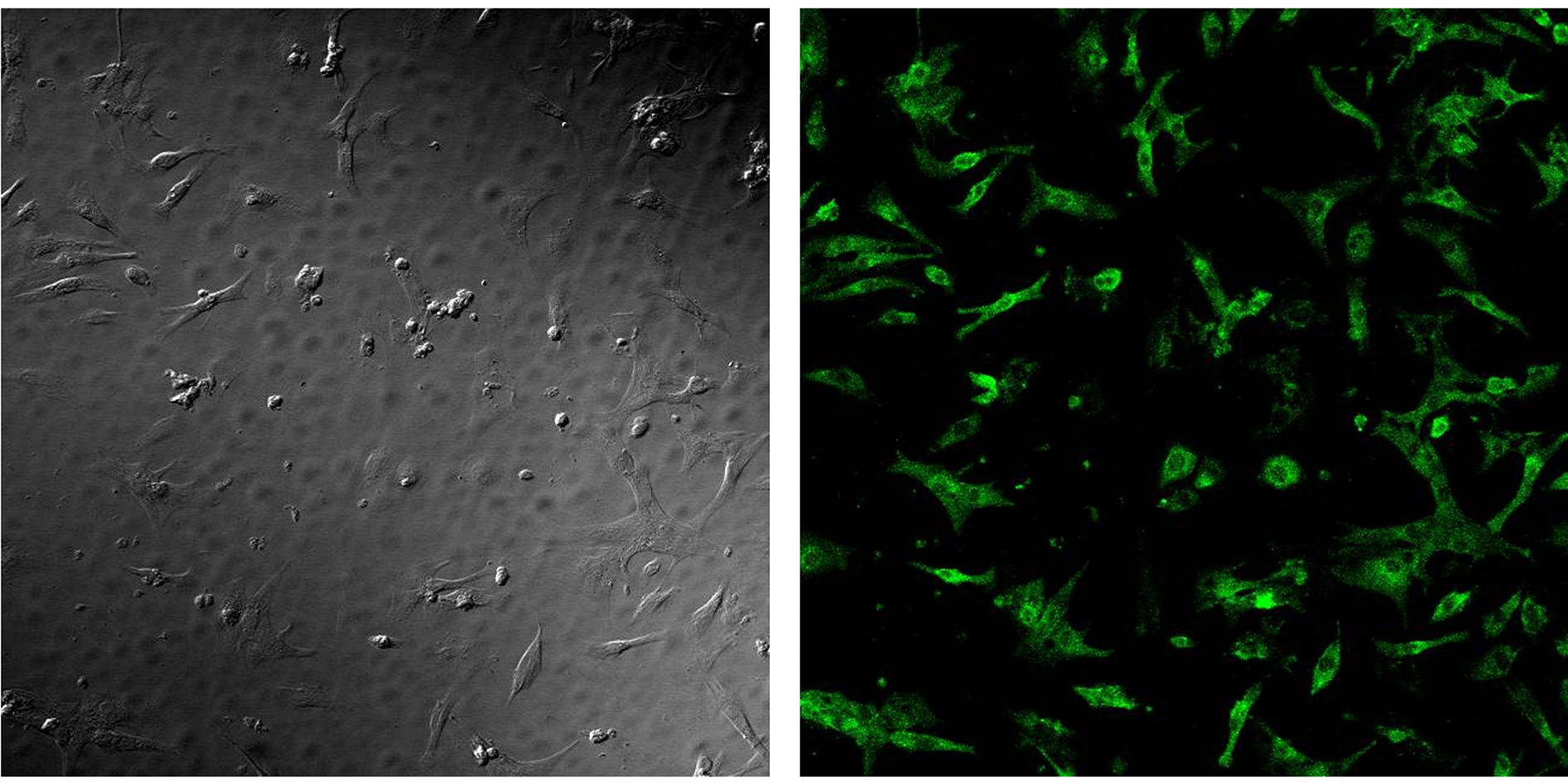

Supplement: Supplementary file 5 — Fig S5 [file JCMM-25-3935-s008.tif]

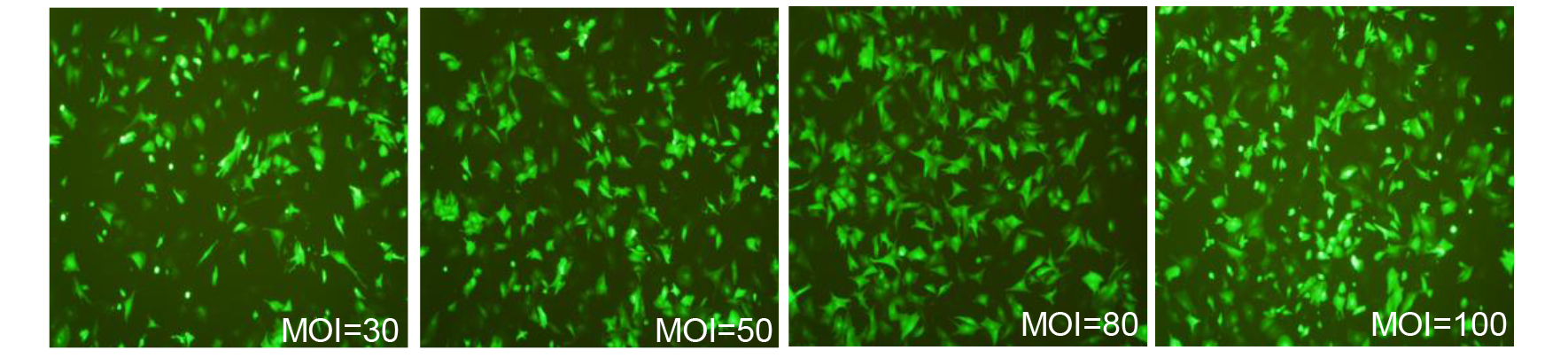

Supplement: Supplementary file 6 — Fig S6 [file JCMM-25-3935-s001.tif]
