# Supplementary material for: Kir6.1 improves cardiac dysfunction in diabetic cardiomyopathy via the AKT‐FoxO1 signalling pathway
Source: J Cell Mol Med. 2021 Feb 6;25(8):3935–49. doi: 10.1111/jcmm.16346 (PMC8051713; doi:10.1111/jcmm.16346)
Supplement: Supplementary file 7 — Table S1 [file JCMM-25-3935-s007.docx]

**Supplemental Table 1.** The data regarding food intake and caloric intake for each experimental group.

| Variables | Regular diet (D12450J) | HFD (D12492) |
| --- | --- | --- |
| Food intake, g/week | 22.5±1.3 | 22.4±0.7 |
| Caloric intake, kcal/week | 86.4±5.0 | 116.7±3.6 |
