# Supplementary material for: Kir6.1 improves cardiac dysfunction in diabetic cardiomyopathy via the AKT‐FoxO1 signalling pathway
Source: J Cell Mol Med. 2021 Feb 6;25(8):3935–49. doi: 10.1111/jcmm.16346 (PMC8051713; doi:10.1111/jcmm.16346)
Supplement: Supplementary file 8 — Table S2 [file JCMM-25-3935-s006.docx]

After 8 h of infection, the medium was changed to fresh DMEM containing serum. Then the 0.4% trypan blue solution was used for cell staining. The total number of four-grid cells and blue-stained cells were observed and recorded at 100× magnification under a microscope (Olympus, Tokyo, Japan). The living cell rate = (total number of four cells-total number of blue-stained cells) / total number of four-grid cells × 100%. The cell viability in Ad-C group had no difference from that of Ad-Kir6.1 group (Supplemental Table 2).

**Supplemental Table 2**. Comparison of cell viability in Ad-C group and Ad-Kir6.1 group (Mean ± SD)

| Group | Independent experiments | The living cell rate (%) |
| --- | --- | --- |
| Ad-C | 5 | 0.91±0.98 |
| Ad-Kir6.1 | 5 | 0.92±1.07 |

*P*>0.05. Original magnification: 100×.
