# Supplementary material for: Kir6.1 improves cardiac dysfunction in diabetic cardiomyopathy via the AKT‐FoxO1 signalling pathway
Source: J Cell Mol Med. 2021 Feb 6;25(8):3935–49. doi: 10.1111/jcmm.16346 (PMC8051713; doi:10.1111/jcmm.16346)
Supplement: Supplementary file 9 — Table S3 [file JCMM-25-3935-s003.docx]

**Supplemental Table 3.** Quantitative polymerase chain reaction primers used in this study

| Primer Name | Forward/Reverse | Sequence (5'-3') |
| --- | --- | --- |
| Kir6.1 | Forward | AGCTGGCTGCTCTTCGCTATCA |
| Kir6.1 | Reverse | CCCTCCAAACCCAATGGTCACT |
| 36β4 | Forward | CAGAGGTGCTGGACATCACAGAG |
| 36β4 | Reverse | GGCAACAGTCGGGTAGCCAATC |
